# Empagliflozin Improves Post-Infarct Heart Failure Through Fibroblast Growth Factor-21 and Ketone Body Oxidation Pathway

**DOI:** 10.3390/cells15161478

**Published:** 2026-08-18

**Authors:** Dao-Fu Dai, Ines Martins, Nastaran Daneshgar, Meng Gao, Benjamin Rodriguez, Mohd Mabood Khan, Antentor Hinton, Peter A. Crawford, Chad Grueter

**Affiliations:** 1 Cardiovascular and Renal Pathology Division, Department of Pathology, School of Medicine, Johns Hopkins University, 632E Ross Building, 720 Rutland Ave, Baltimore, MD 21205, USA; 2 Abboud Cardiovascular Research Center, Department of Internal Medicine, Division of Cardiovascular Medicine, Carver College of Medicine, University of Iowa, Iowa City, IA 52242, USA; 3 Department of Pathology, University of Cincinnati College of Medicine, Cincinnati, OH 45267, USA; 4 Department of Molecular Physiology & Biophysics, Vanderbilt University School of Medicine, Nashville, TN 37232, USAantentor.o.hinton.jr@vanderbilt.edu (A.H.J.); 5 Department of Medicine, and Department of Biochemistry, Molecular Biology, and Biophysics, University of Minnesota, Minneapolis, MN 55455, USA; crawforp@umn.edu; 6 Fraternal Order of Eagles Diabetes Research Center, Carver College of Medicine, University of Iowa, Iowa City, IA 52242, USA

**Keywords:** sodium-glucose cotransporter-2 (SGLT2) inhibitors, Empagliflozine, myocardial infarction, heart failure, beta-hydroxybutyrate, ketone body oxidation, fibroblast growth factor-21

## Abstract

**Background:** Sodium-glucose cotransporter-2 (SGLT2) inhibitors improve outcomes in heart failure, but the mechanisms remain incompletely understood. Metabolic remodeling has been proposed as a key mediator. **Methods and Results:** Myocardial infarction (MI) was induced in cardiomyocyte-specific BDH1 knockout (BDH1-KO) wild-type (WT) mice and in liver-specific Fibroblast Growth Factor-21 knockout (FGF21-KO) mice. Following confirmation of reduced ejection fraction (EF), mice were randomized to empagliflozin (Empa, 10 mg/kg/day) or saline. After 4 weeks, untreated WT mice demonstrated progressive systolic dysfunction (ΔEF: −11.6 ± 6.3%), whereas Empa-treated WT mice showed significant improvement (ΔEF: 9.9 ± 4.3%). This benefit was completely abolished in BDH1-KO mice (ΔEF: −10.5 ± 2.8%) or FGF21-KO mice, suggesting that FGF21 regulation and cardiomyocyte ketone oxidation are required for Empa cardioprotection. In WT and hepatocyte-specific FGF21-KO mice, 1 week of Empa treatment increased cardiac BDH1 expression in WT but not FGF21-deficient mice. In human iPSC-cardiomyocytes, FGF21 induced BDH1 expression, whereas Empa had no direct effect on BDH1. In HepG2 liver cells, Empa increased both FGF21 and BDH1 expression. **Conclusions:** Empa activates the liver–heart metabolic axis. Loss of cardiomyocyte BDH1 or FGF21 production by the liver abolishes Empa-mediated improvement in post-MI cardiac function, identifying FGF21/ketone metabolism as a key mechanism of SGLT2 inhibitor cardioprotection.

## 1. Introduction

Sodium-glucose cotransporter-2 (SGLT2) inhibitors were originally developed as glucose-lowering agents for the management of diabetes mellitus (DM) [1]. Recent clinical trials have revealed a paradigm shift in their clinical utility, demonstrating robust cardioprotective and nephroprotective [2] benefits that extend beyond glycemic control. This is particularly evident in the treatment of heart failure (HF) [3], where SGLT2 inhibitors have consistently reduced the risk of hospitalization and cardiovascular mortality, regardless of the patient’s baseline diabetes status.

The known mechanism of SGLT2 inhibitors is to block SGLT2-dependent glucose reabsorption primarily in the proximal convoluted tubules of the kidney, where SGLT2 is abundant. By inhibiting this transporter, SGLT2 inhibitors induce glycosuria and natriuresis, leading to secondary hemodynamic effects including reduced preload, lowered blood pressure, and improved arterial stiffness. Interestingly, SGLT2 inhibitors confer profound cardioprotective benefits despite the widely accepted fact that the heart primarily expresses the SGLT1 isoform rather than SGLT2 [4]. This discrepancy suggests that the drug’s cardioprotective effects are likely mediated through systemic, pleiotropic mechanisms rather than direct myocardial interaction.

Fibroblast growth factor 21 (FGF21) is a stress-responsive metabolic hormone primarily generated by the liver that is essential for maintaining systemic energy balance. FGF21 is expressed in skeletal muscle, adipose tissue, pancreas, and heart, where it has tissue-specific autocrine and paracrine effects, even though the liver is the main endocrine source of circulating FGF21 [5]. Hepatic FGF21 protects against hepatic lipotoxicity and promotes fatty acid β-oxidation, ketogenesis, and metabolic adaptability. It is induced by fasting, ketogenic diets, endoplasmic reticulum (ER) stress, and mitochondrial dysfunction [6]. In skeletal muscle, FGF21 acts as a stress-induced myokine, increasing lipid utilization and insulin sensitivity. Importantly, cardiac FGF21 functions as an endogenous cardioprotective factor in response to ischemia, pressure overload, oxidative stress, and mitochondrial injury [7]. FGF21 may play a mediating role in the metabolic and cardioprotective benefits of SGLT2 inhibitors [8].

Ongoing research has proposed a multifactorial landscape of secondary benefits, ranging from attenuating oxidative stress and systemic inflammation to inhibiting maladaptive cardiac remodeling and fibrosis. Among these, metabolic reprogramming has emerged as a cornerstone of the hypothesis. Under physiological conditions, the healthy myocardium preferentially relies on fatty acid oxidation for efficient ATP synthesis [9]. However, in the context of heart failure, this metabolic flexibility is severely impaired.

Recent clinical trials have shown that SGLT2 inhibitors have shown a beneficial effect across the spectrum of left ventricular ejection fraction (LVEF), ranging from heart failure with reduced EF (HFrEF) to preserved EF (HFpEF) [10,11]. One potential mechanism is by inducing a unique metabolic shift by increasing circulating ketone body levels, particularly β-hydroxybutyrate. Unlike fatty acids, which require higher oxygen consumption for oxidation, ketone bodies represent a “superfuel” that can be utilized by the failing myocardium, potentially enhancing the efficiency of cardiac energetics and mitigating the deleterious effects of metabolic starvation frequently seen in HF [12]. Therefore, it is critical to clarify the precise mechanistic link between SGLT2 inhibition and myocardial metabolism, which may unlock new therapeutic avenues for heart failure management.

We hypothesize that the augmentation of myocardial ketone body utilization serves as a critical mechanism driving the restoration of cardiac function in HFrEF. While the pleiotropic effects of SGLT2 inhibition are well-documented, the mechanism underlying this metabolic transition remains inadequately understood. In this study, we aim to elucidate how the SGLT2 inhibitor Empagliflozin (Empa) drives adaptive metabolic remodeling by activating the fibroblast growth factor-21 (FGF-21) axis and the ketone body oxidation pathway. We focus on the regulatory role of β-hydroxybutyrate dehydrogenase-1 (BDH1), the mitochondrial enzyme that catalyzes the rate-limiting step of ketone body oxidation—the conversion of β-hydroxybutyrate to acetoacetate. We propose that Empa-induced upregulation of the FGF-21/BDH1 signaling cascade [8,13] enhances the heart’s capacity to switch from oxygen-intensive fatty acid oxidation to the more energetically efficient utilization of ketone bodies. By examining this pathway, we seek to determine whether the metabolic transition towards the myocardium processing ketone bodies as an alternative fuel is a downstream mechanism underlying the functional improvement observed in post-infarct HFrEF following SGLT2 inhibitor therapy.

## 2. Method

### 2.1. Animal Model of Myocardial Infarction

Animal experiments received approval from the Institutional Animal Care and Use Committees (IACUC) at the University of Iowa (0082063) and Johns Hopkins University (M023M236). All protocols adhered to the NIH Guide for the Care and Use of Laboratory Animals and the European Parliament Directive 2010/63/EU. Mice were maintained in a temperature-controlled facility under a 12 h light/dark cycle with ad libitum access to food and water.

The myocardial infarction (MI) studies exclusively utilized 8- to 12-week-old male C57BL/6J mice, including TnT-Cre-BDH1^fl/fl^ (cardiomyocyte-specific BDH1 KO) and BDH1floxed without Cre (wild-type littermate), Alb-Cre FGF21^fl/fl^ (liver-specific FGF21 KO) and those without Cre (wild-type littermate). Myocardial infarction (MI) was induced in mice (~60 total, including all groups) using an established, ventilator-assisted technique involving ligation of the left anterior descending artery following a left intercostal thoracotomy [14]. In brief, anesthesia was induced via inhalation of 2% isoflurane. Animals were mechanically ventilated in positive end-expiratory pressure (PEEP) mode with a stroke volume of 200 µL and a respiratory rate of 200 strokes per minute. Following a skin incision to expose the left pectoralis major muscles, the muscle layers were divided, and a thoracotomy was performed through the intercostal space. The heart was gently exteriorized through the incision, and the left anterior descending (LAD) coronary artery was ligated using a 10-0 Prolene suture (Ethicon, Cincinnati, OH, USA). The suture was placed approximately 1 mm distal to the left atrial appendage, immediately downstream of the major left coronary artery bifurcation. The thoracotomy was closed by securing the third and fourth ribs together with 1–2 interrupted 5/0 chromic catgut absorbable sutures, followed by skin closure using a continuous 6/0 Prolene suture. Upon extubation and detachment from the ventilator, the mice immediately re-established spontaneous respiration.

The inclusion criteria: LVEF < 0.65 after surgery (Normal LVEF by 2D echo is approximately 0.8–0.9). To minimize heterogeneity and eliminate potential confounders (surgery-related mortality), mice displaying an EF < 20%, exhibiting severe bradycardia (heart rate < 400 beats/min), or that died within 5 days post-surgery were excluded.

### 2.2. Echocardiography

Post-surgical cardiac function was assessed in conscious mice using transthoracic echocardiography (Vevo 2100, VisualSonics, Toronto, ON, Canada) to measure left ventricular dimensions and performance. Cardiac images were acquired using a 30 MHz linear array transducer, capturing parasternal short- and long-axis views at a frame rate of 180–250 Hz. Offline image analysis was performed utilizing the Vevo 2100 software platform (version 1.5). Left ventricular (LV) dimensions and functional parameters were evaluated using longitudinal M-mode echocardiography. LV ejection fractions were measured using 2D endocardial tracings, at baseline (24–48 h after surgery) and at endpoint (after treatment). The changes in EF were calculated by the endpoint EF minus baseline EF.

### 2.3. Human Induced Pluripotent Stem Cell-Derived Cardiomyocyte (hiPSC-CM) Differentiation, Purification, and Transfection

Human induced pluripotent stem cells (hiPSCs) were maintained on Matrigel-coated plates (Corning Life Sciences, New York, NY, USA) in a mixture of 50% serum-free medium (SFM, ATCC, Manassas, VA, USA) and 50% mTeSR plus medium (STEMCELL, Vancouver, BC, Canada). For cardiac differentiation, hiPSCs were dissociated using a gentle cell dissociation reagent (from STEMCELL, Vancouver, Canada) and seeded onto 12-well plates. Upon reaching 95% confluency, differentiation was initiated by modulating the Wnt pathway. Briefly, cells were treated with 5 µM CHIR99021 in RPMI 1640 medium supplemented with 2% B27 minus insulin (differentiation medium) on day 0, with supplementary medium additions on days 1 and 2. On day 3, the medium was replaced with differentiation medium containing 5 µM IWR-1. Cells were refreshed with insulin-free differentiation medium on day 5 and transitioned to standard CM culture medium (RPMI 1640 with 2% B27) on day 7. To enrich the cardiomyocyte population, metabolic purification was performed on day 9 using glucose-free RPMI 1640 medium supplemented with 2% B27 and sodium L-lactate (Sigma-Aldrich, St.Louis, MO, USA). From day 14 onward, the enriched hiPSC-CMs were cultured in the presence of tri-iodo-L-thyronine (T3; 100 nmol/L) and dexamethasone (1 μmol/L) to promote structural and functional maturation. The hiPSC-CMs were utilized for downstream experiments between days 22 and 30 post-differentiation. Briefly, hiPSC-CMs were treated with FGF21 (1, 25, or 50 ng/mL) or Empa (10 μM) for 48 h, followed by protein extraction in cold RIPA buffer supplemented with protease/phosphatase inhibitors.

### 2.4. HepG2 Cell Culture, RNA and Protein Extraction and Quantitative PCR

HepG2 liver cells were grown in Eagle’s Minimum Essential Medium (EMEM) supplemented with glutamine and 10% fetal bovine serum (FBS), 100 U/mL penicillin, and 100 µg/mL streptomycin, at 37 °C in a humidified incubator with 5% CO_2_. Cells were treated with Empa (0 (saline control), 10 or 50 μM) for 8, 24, and 48 h, followed by RNA or protein extraction. For RNA, total RNA was extracted from HepG2 cells using ice-cold TRIzol, followed by RNA extraction with an RNA extraction kit. cDNA Synthesis: Reverse transcription was performed using a high-capacity cDNA reverse transcription kit. Quantitative PCR was performed using SYBR Green-based quantitative RT-PCR for FGF21 and GAPDH (loading control). The relative expression was calculated using the delta-delta Ct method. Protein extraction was performed using a cell scraper in ice-cold radioimmunoprecipitation assay (RIPA) buffer containing protease and phosphatase inhibitors.

### 2.5. Western Blotting

Heart ventricles, liver, or cells were homogenized in RIPA lysis buffer supplemented with Complete Mini protease inhibitor and phosphatase inhibitor cocktail. An equal amount of protein was loaded onto an SDS-PAGE gel and then underwent electrophoresis. The separated proteins were transferred to PVDF membranes, which were then blocked with a 4% bovine serum albumin (BSA) solution in TBS for 1 h at room temperature. Following blocking, the membranes were incubated overnight at 4 °C with primary antibodies diluted in the 4% BSA blocking solution. The primary antibodies include BDH1 (1:2000, Proteintech 15417-1-AP, Rosemont, IL, USA), FGF21 (Invitrogen JA10-97; 1:1000, Thermo Fisher Scientific, Waltham, MA, USA) and β-actin (1:500, Santa Cruz Biotechnology, sc-47778, Dallas, TX, USA), GAPDH (1:500 Santa Cruz Biotechnology, sc-32233). After a triple wash with TBS-Tween, incubation with the corresponding secondary antibodies was performed for 1 h, followed by another triple wash with TBS-Tween. The membranes were then visualized using SuperSignal West Femto (Thermo Fisher Scientific).

## 3. Result

### Empagliflozin(Empa) Improves Post-Myocardial Infarction (MI) Systolic Function by Promoting Cardiac Ketone Body Oxidation

**Figure 1:**
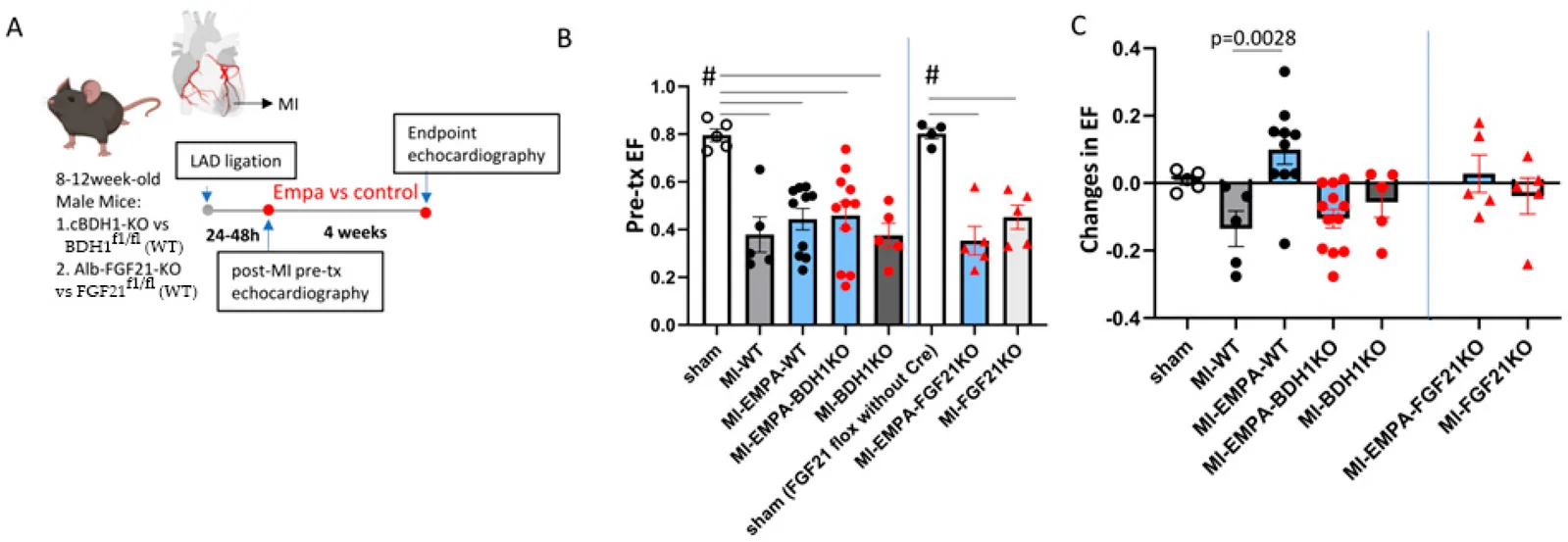
(**A**) Experimental Design. 8–12-week-old male C57Bl6/J mice. Two sets of experiments: (1) Cardiomyocyte-specific BDH1 KO (BDH1^fl/fl^ with TnT-Cre vs. No Cre controls), (2) Liver-FGF21 KO (FGF21^fl/fl^ with Albumin Cre vs. no Cre control). (**B**) Pre-treatment echocardiography was performed 24-48 h post-mid LAD ligation to confirm MI. (**C**) Changes in Ejection Fraction (ΔEF) calculated by EF ~4 weeks after treatment and pre-treatment. Second echocardiography in the same mice was performed ~4 weeks after Empa or saline treatment in wild-type (WT; BDH1^fl/fl^ or FGF21^fl/fl^ without Cre) and c-BDH1.KO (TnT-Cre) and FGF21.KO (Alb-Cre) male mice; *n* = 5–12; ANOVA post hoc Dunn multiple comparison tests; # *p* < 0.05 vs. each of the remaining groups. Only significant tests were shown.

**Figure 2:**
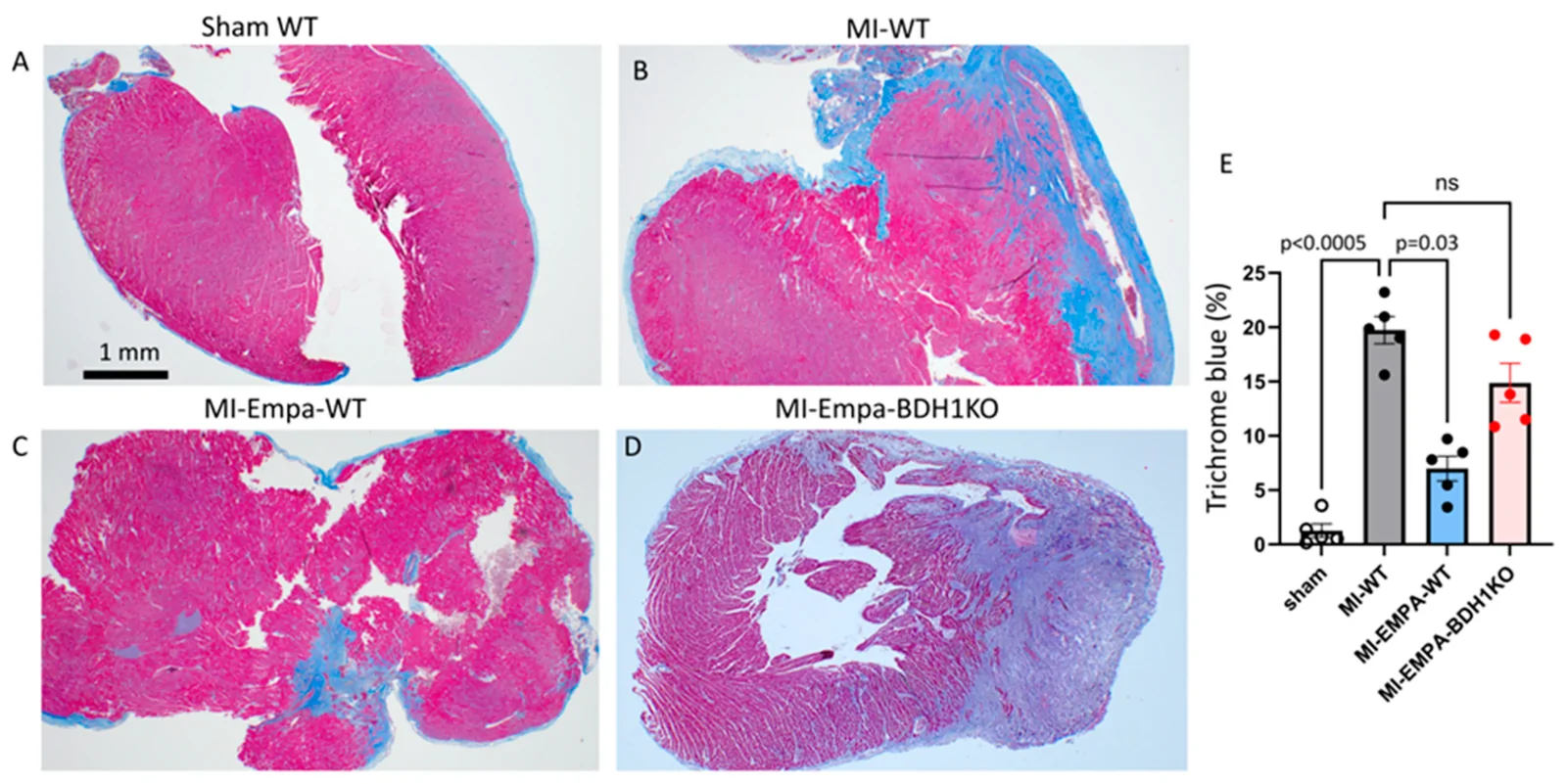
Representative Masson Trichrome staining. (**A**) Sham in WT mice, (**B**) MI in WT, (**C**) MI in WT treated with Empa, (**D**) MI in c-BDH1.KO treated with Empa, (**E**) Relative area of Trichrome blue staining (%); *n* = 5; ANOVA post hoc Dunn multiple comparison tests; ns: not significant.

Experiments evaluating the effect of Empa following MI are summarized in Figure 1A, which outlines the mouse experimental design. Myocardial infarction was induced in both wild-type (BDH1^fl/fl^) and cardiomyocyte-specific BDH1 knockout (BDH1^fl/fl^, TnT-Cre) mice using mid left anterior descending (LAD) artery ligation. Successful MI induction was confirmed by conscious echocardiography, performed 24 to 48 h post-surgery, which confirmed the successful induction of MI, characterized by a marked reduction in left ventricular ejection fraction (LVEF). Following MI, mice were randomized to receive either Empa (10 mg/kg/day) or a saline vehicle. Baseline echocardiography showed no significant difference in the post-MI LVEF among different experimental groups, confirming effective randomization (Figure 1B). Echocardiography was repeated approximately 4 weeks post-treatment, with sonographers blinded to the treatment/genotypes to minimize bias. The wild-type MI mice treated with saline exhibited a progressive decline in LVEF, with an average reduction of −11.6% ± 6.3% (Figure 1C), indicating the well-known post-MI adverse ventricular remodeling leading to heart failure with reduced ejection fraction (HFrEF). In contrast, Empa-treated wild-type MI mice demonstrated a significant improvement in systolic function, achieving an average EF increase of +9.94% ± 4.3% relative to baseline post-MI measurements. However, Empa treatment did not improve cardiac function in cardiomyocyte-specific BDH1 knockout mice, which demonstrated a mean decline in LVEF (−10.5% ± 2.8%), comparable to the deterioration observed in saline-treated wild-type control mice. Pathological examinations confirm the findings of echocardiography. Masson Trichrome staining reveals infarct scar and replacement fibrosis in WT mice receiving MI surgery (Figure 2B), ~20% compared with minimal fibrosis in WT mice receiving sham surgery (Figure 2A,B,E, *p* = 0.0004 MI-WT vs. sham-WT). Empa treatment significantly attenuated the relative area of fibrosis (Figure 2B,C,E, *p* = 0.03 for Empa-MI-WT vs. MI-WT). In contrast, Empa treatment did not improve fibrosis (infarct scar) in BDH1 knockout mice (Figure 2D), indicating that the protective effect of Empa on post-MI adverse ventricular remodeling requires intact BDH1. This emphasizes the critical role of BDH1 in ketone body oxidation as one downstream mechanism of Empa effect.

**Figure 3:**
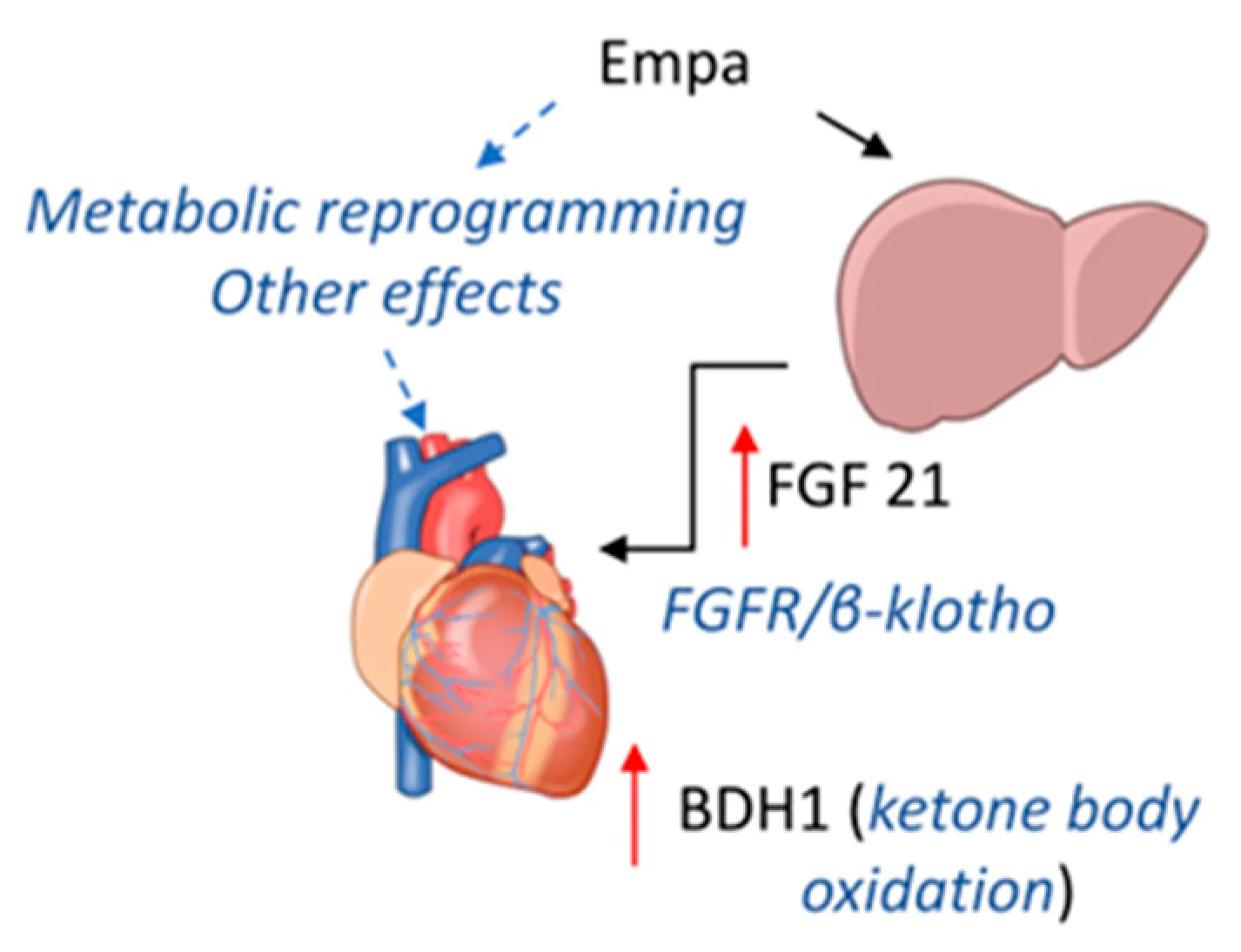
Mechanistic Diagram. Empa induced liver FGF21, a circulating hormone that upregulates cardiomyocytes’ BDH1, a critical enzyme in ketone body oxidation.

We propose that enhanced ketone body utilization is one of the underlying mechanisms for improved cardiac function in HF with reduced ejection fraction (HFrEF). To test this hypothesis, we examined the metabolic remodeling by Empa, focusing on the fibroblast growth factor-21 (FGF-21) and ketone body oxidation pathways [8,13]. Specifically, we investigated beta-hydroxybutyrate dehydrogenase-1 (BDH1), the rate-limiting enzyme in ketone metabolism. FGF21 is a hepatokine primarily secreted by hepatocytes. Figure 3 illustrates our proposed mechanism for the cardioprotective effects of SGLT2 inhibitors (the italicized parts were not directly tested in this study). To examine its role in this context, we induced MI in hepatocyte-specific FGF21.KO (FGF21^fl/fl^ with Alb-Cre) and randomly assigned to two groups: 5 receiving Empa (10 mg/kg/d, ip) and 5 receiving saline. No significant changes in EF were observed between the two groups. These findings suggest that Empa does not improve post-MI heart failure in the absence of FGF21. Additionally, FGF21 KO alone did not significantly affect post-infarct heart failure progression.

**Figure 4:**
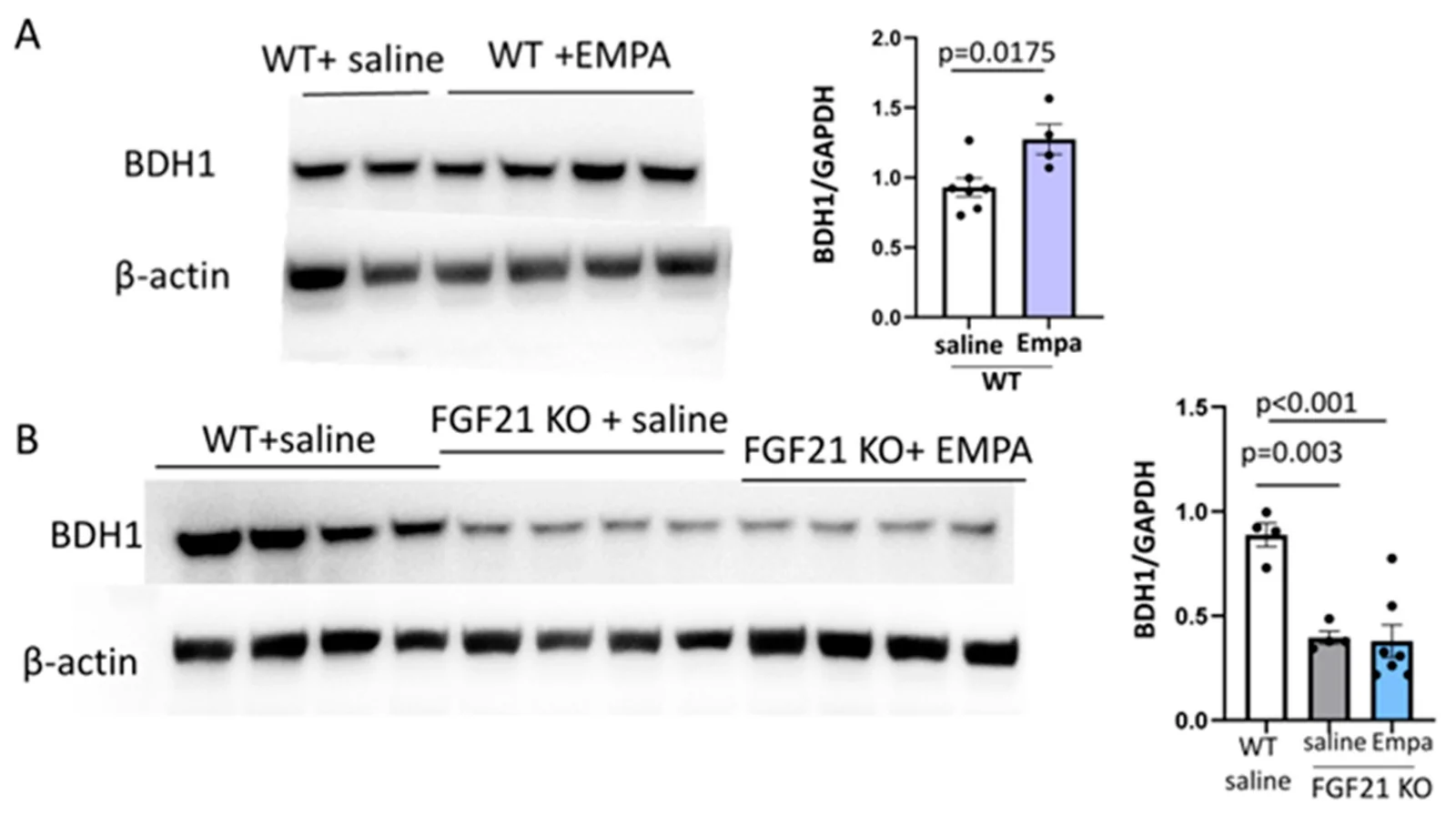
Cardiac BDH1 upregulation mediated by FGF21. (**A**) WT or (**B**) Alb-FGF21-KO mice treated with Empa (10 mg/kg/d, one week) or saline; *n* = 4–7, by two-sample *t*-tests or ANOVA Sidak post hoc tests.

This Empa-induced, FGF21-dependent hepatic axis provides a critical metabolic adaptation to restore energy balance in energy-starved cardiomyocytes. To rigorously evaluate the hypothesis that Empa upregulates cardiac BDH1 via an FGF21-dependent mechanism, we conducted both in vivo and in vitro assessments. To determine if hepatic FGF21 mediates the cardiac upregulation of BDH1, wild-type (WT) and *Alb-FGF21* KO mice were treated with a daily gavage of Empa (10 mg/kg/d) for one week. In WT mice (FGF21^fl/fl^ without Alb-Cre), Empa treatment resulted in a significant ~20% increase in cardiac BDH1 protein levels (Figure 4). In contrast, the Empa-induced increase in cardiac BDH1 was absent in *Alb-FGF21* KO mice, suggesting that hepatic FGF21 is required for this downstream increase in cardiac BDH1 expression. Our findings support a model in which the FGF21-BDH1 signaling axis is an essential step in promoting β-hydroxybutyrate oxidation, thus providing crucial ketone bodies as an alternative energy substrate for the energy-starved, failing heart (Figure 4).

**Figure 5:**
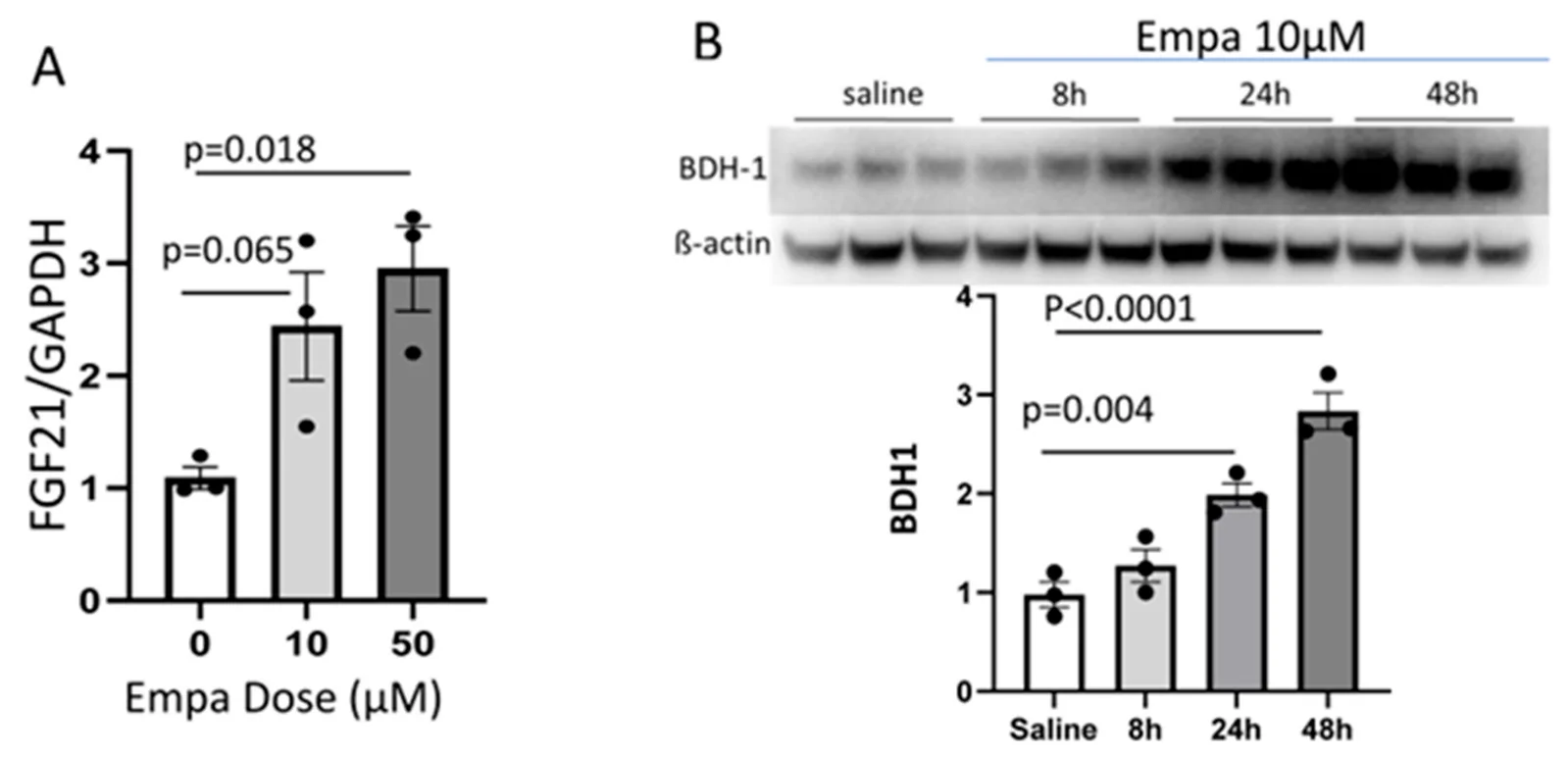
HepG2 human liver cell lines (**A**) Quantitative PCR of FGF21 in liver cell lysate, 24 h after Empa treatment. (**B**). Empa 10μM increased BDH1 over time. *n* = 3–4; ANOVA post hoc tests.

**Figure 6:**
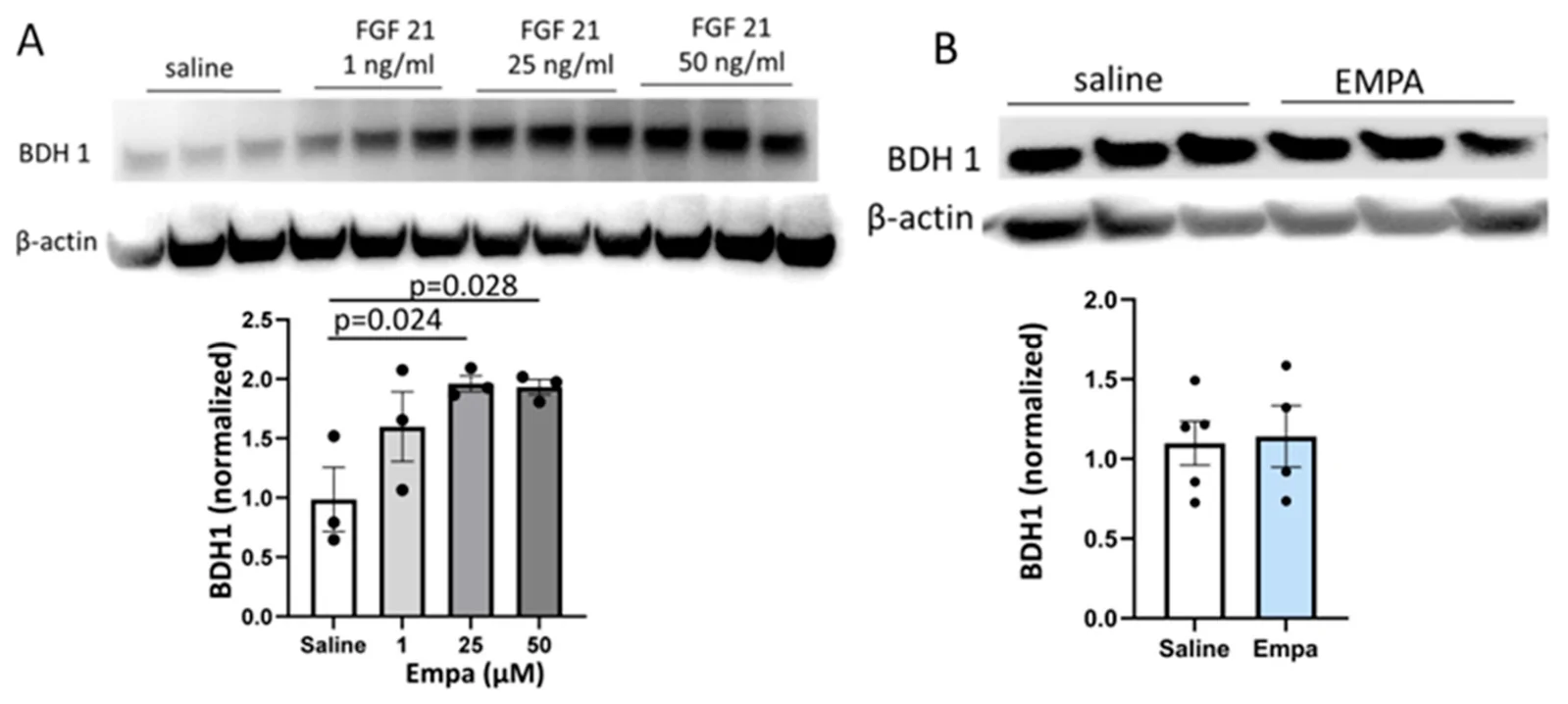
Human induced pluripotent-stem-cell derived cardiomyocytes (iPS-CM) (**A**). FGF21 increased BDH1 in a dose-dependent manner. (**B**). Empa (10 µM, 48 h) had no significant direct effect on cardiomyocyte BDH1; *n* = 3–5, ANOVA, Sidak post hoc tests, or Student *t*-test.

To reinforce the in vivo findings and clarify cell-specific responses, we treated human-induced pluripotent stem cell-derived cardiomyocytes (iPSC-CMs) and HepG2 liver cells with FGF21 or Empa. As shown in Figure 5, in HepG2 liver cells, Empa (10 μM) significantly increased BDH1 protein expression. Concurrently, Empa exposure induced a dose-dependent upregulation of FGF21 in these HepG2 cells, confirming that Empa directly acts on liver cells to trigger the FGF21/BDH1 axis. However, direct treatment with Empa (10 μM) in cardiomyocytes did not affect BDH1 levels (Figure 6B), suggesting that Empa did not directly upregulate BDH1 within cardiomyocytes. In contrast, exposure of iPS-CM to escalating doses of FGF21 demonstrated a dose-dependent upregulation of BDH1, indicating that cardiomyocyte BDH1 expression is regulated by FGF21 (Figure 6A).

## 4. Discussion

Our study identifies an essential, non-canonical metabolic signaling axis through which empagliflozin (Empa) exerts cardioprotective effects in the setting of myocardial infarction (MI), consistent with a meta-analysis showing beneficial effects in post-MI patients [15]. We demonstrate that the improvement of systolic function following post-infarct heart failure is likely not a direct result of Empa action on the cardiomyocytes’ metabolism, but rather the consequence of a systemic liver-cardiac relay through an FGF21/BDH1 axis. These findings extend current understanding of SGLT2 inhibitor biology by identifying the liver as a critical metabolic relay that promotes adaptive myocardial ketone utilization during post-infarction remodeling.

A pivotal aspect of our study is the elucidation of the requirements for Empa-induced cardiac metabolic reprogramming. Through in vitro experiments utilizing iPSC-CMs and HepG2 cells, we demonstrated that Empa does not directly upregulate BDH1 in cardiomyocytes. Instead, Empa appears to act directly on hepatic cells to trigger the upregulation of FGF21, which then exerts paracrine effects on the hepatocyte and endocrine effects on cardiomyocytes to upregulate BDH1, thereby augmenting the ketone body oxidation pathway and preserving myocardial energetics by providing alternative super-fuels in the injured myocardium [16]. This finding is significant as it suggests SGLT2 inhibitors as metabolic regulators that utilize the liver as a functional relay station to fuel energy-starved cardiomyocytes.

Increasing evidence suggests that SGLT2 inhibitors increase ketone metabolism. Previous experimental and clinical research have demonstrated that SGLT2 inhibitors enhance circulating ketone body concentrations and enhance myocardial energy efficiency [17,18]. Ketone bodies, such as β-hydroxybutyrate, have been postulated as a “super fuel” due to their higher efficiency in producing ATP per molecule of oxygen used compared to fatty acids [17]. This advantage may be especially crucial in ischemic and failing hearts. Furthermore, transcriptomic and metabolomic investigations have shown that SGLT2 inhibitor medication increases the expression of ketone consumption genes such as BDH1, OXCT1 (SCOT), and mitochondrial oxidative enzymes [19]. These findings support the idea that SGLT2 inhibitors enhance a metabolic shift toward ketone oxidation rather than just increasing ketone availability.

The clinical success of SGLT2 inhibitors in heart failure has long been attributed to various hemodynamic and metabolic benefits, yet the precise molecular mechanisms remain incompletely understood. The heart primarily expresses the SGLT1 isoform, with extremely low SGLT2 expression in adult cardiomyocytes under physiological conditions [4]. A recent study in human hearts showed a significant upregulation of SGLT2 in diabetic failing hearts and in response to high glucose [20]. This may suggest beneficial effects via direct inhibition of SGLT2 in the context of diabetic cardiomyopathy. However, the cardioprotective effect of Empa in HFrEF was preserved in mice with global knockout of SGLT2 [21], emphasizing the “off target” pleiotropic effect, such as through metabolic reprogramming. This is further reinforced by the protective effects of Empa on both cardiovascular and renal outcomes independent of diabetes in the EMPEROR clinical trial [3]. Our data suggest one “off target” mechanistic explanation. The failure of Empa to rescue cardiac function in cardiomyocyte-specific BDH1-knockout mice highlights the critical role of ketone body oxidation in the post-infarct failing heart [12,16]. We further showed the dependence on hepatic FGF21, as liver-specific FGF21KO also abolished the cardioprotective effect of Empa. This suggests that the liver acts as a metabolic transducer. By producing FGF21 in response to Empa, the liver appears to initiate a broader metabolic adaptation that ultimately enhances the heart’s ability to meet its energetic demands during the progression of heart failure. By demonstrating that Empa fails to directly upregulate BDH1 in iPSC-derived cardiomyocytes while robustly inducing FGF21 and subsequent BDH1 expression in liver cells, our findings elucidate the direct versus indirect signaling pathways of SGLT2 inhibition.

FGF21 is a circulating hormone primarily produced by the liver and has emerged as a central regulator of systemic metabolism during fasting, nutrient deprivation, and metabolic stress [22]. Beyond its established roles in glucose homeostasis, lipid metabolism, and insulin sensitivity, accumulating evidence indicates that FGF21 also participates in cardiovascular adaptation. Emerging studies suggest that FGF21 activation may offer therapeutic benefit in metabolic disorders, particularly in patients with metabolic dysfunction-associated steatotic liver disease [23]. As a stress response hormone, FGF21 enhances insulin sensitivity and promotes longevity in mice with diet-induced obesity [24]. It triggers metabolic reprogramming with benefits similar to those of lifelong caloric restriction or fasting. Interestingly, FGF21 also plays a critical role in suppressing alcohol dependence [25]. FGF21 activates FGFR1 signaling, which maintains mitochondrial function, regulates oxidative stress and inflammation, inhibits cardiomyocyte apoptosis, and reduces pathological hypertrophy and adverse cardiac remodeling. FGF21 also helps maintain cardiac energy metabolism and function [26]. In adipose tissue, FGF21 increases energy expenditure by promoting glucose absorption, improving insulin sensitivity, and inducing browning of white adipose tissue [27]. Recent evidence suggests the beneficial effect of SGLT2 inhibition through reprogramming.

Our findings suggest that Empa activation of the FGF21-BDH1 axis appears to promote a broader metabolic shift that allows the myocardium to more effectively utilize ketone bodies during periods of energetic stress. Such flexibility may be particularly advantageous following myocardial infarction, where oxygen availability and mitochondrial efficiency are often compromised. The failing heart is characterized as an energy-starved state with defective mitochondrial function. A deficit in mitochondrial substrate flexibility further contributes to adverse remodeling and contractile dysfunction. Our results suggest that Empa acts to unlock an adaptive metabolic state. By augmenting the FGF21-BDH1 pathway, Empa enables the myocardium to transition from fatty-acid reliance to more efficient ketone body utilization [28], especially when oxygen supply is rather limited. Notably, our observation that FGF21 deficiency did not independently worsen the progression of heart failure suggests that this pathway is not merely a homeostatic baseline but a specialized therapeutic response induced by SGLT2 inhibition to bypass metabolic blockade.

These findings reframe SGLT2 inhibitors as central regulators of inter-organ communication, suggesting the liver’s pivotal role in supporting cardiac energetics. This FGF21-dependent mechanism provides a potential explanation for the rapid improvement in cardiac outcomes observed in clinical trials, as this signaling cascade likely operates independently of SGLT2-mediated glucose lowering. The metabolic reprogramming by SGLT2 inhibitors may also be mediated by FGF21-independent mechanisms [8] and the favorable modulation of several signaling pathways, such as mTOR complex I inhibition [29]. Further studies are warranted to elucidate additional mechanisms of these blockbuster drugs, which may interact with AMPK (fasting-like responses) and Peroxisome Proliferator-Activated Receptor Alpha and Gamma pathways (critical in lipid metabolism and energy homeostasis). Future research should determine whether this axis can be modulated in patients with insulin resistance or advanced liver disease, in whom the hepatic response to FGF21 signaling may be impaired. Furthermore, the identification of the FGF21-BDH1 axis provides a platform to explore whether direct activation of this pathway could serve as a secondary therapeutic strategy for patients with HFrEF who are unresponsive to conventional therapies. Taken together, this research establishes that the cardioprotective benefits of empagliflozin are at least partly driven by an FGF21-dependent increase in cardiac BDH1, enhancing the metabolic plasticity of the heart.

Several limitations of this study should be acknowledged. First, these experiments were performed primarily in murine models of post-MI HFrEF and human cell models. Therefore, further validation is required in patient populations and other types of heart failure, such as non-ischemic cardiomyopathies and HFpEF. Second, although our data establish a requirement for both hepatic FGF21 and cardiac BDH1, the detailed signaling mechanisms are incompletely understood. Third, upregulation of FGF21 is expected to increase circulating ketone bodies. Given the complexity of obtaining reliable metabolic flux analysis for both β-hydroxybutyrate and acetoacetate in sick mice, the measurement was not performed. Without this data, our findings support but do not prove every step of our proposed hypothesis. Fourth, although HepG2 cells are widely used, they may differ from primary hepatocytes with respect to metabolic regulation and FGF21 production. Finally, it remains unclear whether similar mechanisms contribute to the beneficial effects observed with other members of the SGLT2 inhibitor class. Addressing these questions will be important for determining the broader translational relevance of this pathway.

## 5. Conclusions

Our findings identify a liver-heart signaling axis that contributes to the cardioprotective actions of Empa following myocardial infarction. Empa enhances the metabolic adaptability of the injured myocardium by promoting BDH1-dependent ketone metabolism in the heart, and this is likely dependent on hepatic FGF21 production. These results not only provide new insight into the potential mechanisms underlying SGLT2 inhibitor therapy but also highlight the broader importance of inter-organ metabolic communication in the pathogenesis and treatment of heart failure. As therapies increasingly move beyond single-organ targets, understanding how the liver and heart cooperate during disease may reveal new opportunities to improve outcomes in patients with cardiovascular disease.

## Data Availability

The original contributions presented in this study are included in the article. Further inquiries can be directed to the corresponding author.
